# Shooter ready? Integrating mental skills training in an advanced sniper course

**DOI:** 10.3389/fpsyg.2023.1198986

**Published:** 2023-06-15

**Authors:** Christian Ytterbøl, Dave Collins, Alan MacPherson

**Affiliations:** ^1^Moray House School of Education and Sport, University of Edinburgh, Edinburgh, United Kingdom; ^2^Department of Leadership and Tactics, Norwegian Military Academy, Norwegian Defence University College, Oslo, Norway

**Keywords:** marksmanship training, peak performance, mental skills package, military, stress management, cognition, mindset

## Abstract

Performance psychology has increased in usage and popularity; however, we contend that within the elite spheres of the military, there is a need for research and development to fit the context and environment. In this study, we describe an explorative case study on the integration of mental skill training techniques to an advanced sniper course in the Norwegian Armed Forces. We evaluate the impact through triangulation and examine results on the course, perceptions of the participants, and observations from the instructors. In addition, we conducted a 1-year follow-up to get participants' experience of translating the skills beyond the course. The results show that the mental skill training package influenced both results and performance in a positive manner; however, as a novel field, further research is warranted to establish a best practice to enhance performance for elite military forces.

## Introduction

In the world of professional sports, refining and integrating psychological skills is often viewed as a logical extension of physical training (e.g., MacNamara et al., 2010; Toth et al., 2020). When it comes to improving human performance, the positive impact of psychological skills interventions is both well-known and accepted (Mahoney et al., 1987; Whelan et al., 1991; Birrer and Morgan, 2010; MacNamara et al., 2010; Macnamara et al., 2016). In addition, there is a considerable body of research that supports the implementation of mental skill training (MST) for performers outside sports who routinely experience stress invoking events, such as first responders and practitioners in the field of emergency medicine (e.g., Cocks et al., 2014; Anton et al., 2017, 2020; Deshauer et al., 2019). Internationally, there remains a gap in the human performance psychology research literature as to how allied MST techniques and practices can best be utilized in the military in general (Jensen et al., 2020). Furthermore, given the range of specialisms within the military, it is important to address each subgroup context and culture specifically (Ytterbøl et al., 2022). Of course, effective performance psychology interventions have been tested and applied in apparently similar environments such as Special Weapons and Tactics (SWAT) teams (cf. Liu et al., 2018; Steingräber et al., 2022). Because of our interest and involvement, we wanted to examine whether MST could be applied to another extreme environment, namely the training of snipers. With reference to marksmanship and its associated psycho-motor-behavioral aspects (e.g., Konttinen et al., 1995, 2007; Laaksonen et al., 2018; Tornero-Aguilera et al., 2021), interesting work has been undertaken to understand and improve shooting skills—albeit in a sport shooting environment. Whether as a rifleman or an athlete, aspects of firing a weapon are motorically alike (Magill and Anderson, 2014), but the decision-making trade-offs bear comparison (stealth vs. engagement). In short, military applications of this skill are markedly different. Sport-related shooting is performed in controlled environments, whereas a marksman/sniper must perform in a highly unpredictable environment, often with a looming threat to own safety (Dougan, 2004; Pegler, 2011). Consequently, it is important to distinguish this context from the one in the current literature, which is closest to it, namely SWAT teams. Table 1 presents a comparison using the Antecedent, Behavior, Consequences model (Yoman, 2008).

**Table 1:** ABC contrast between SWAT teams and snipers.

	**Antecedence**	**Behavior**	**Consequence**
SWAT team	External orders, very specific to mission. Communication with command continuously. The environment around the objective is secured. Support systems integrated and short time on target. Tactical superiority/lower risk to own personnel.	Members of a team, rehearsing an already well-known drill. Decisions under pressure, but driven by command, SOP's, or shared mental models.	Debrief against a set structure. On to the next job. Diffusion of responsibility: Clear justification to general role and specific action. Inquiry if shots fired as a normal procedure.
Military snipers	External orders, very unspecific on details. Mission Command. Tactical decisions based on the intention of the commander, on the fly in an already pressured situation. Pre-planned missions with longer planning time or immediate response. Communication with command sporadically (set parameters). Behind enemy lines (drones, Arty etc, own forces, opposing forces). Unsupported. Long duration of the mission. Extreme risk to own personnel.	A team structure, but often operating in pairs, shooter and spotter. Extreme individual responsibility.	Multiple objectives inside a mission can change based on priorities, decided by the snipers. Delegation of frontline battlefield command: Concentration of responsibility: My decision, my consequence, my action. Snipers are always targets on the battlefield. Infiltration, execution, and exfiltration

As shown, the contexts and consequent challenges differ considerably, and perhaps one of the largest differences for a sniper vs. in a SWAT team is the volume of individual decision-making, based on the intention of the mission and what is happening in the battlespace.

## The context of the sniper course

As the context described in this study, the Norwegian Army sniper course is 7 weeks in duration. The course is built around sniper tactics and techniques. In short, in addition to shooting from several weapon platforms in a variety of different contexts, the other key skills include navigation, stalking, observing, plus using both analog and technological methods to measure wind, distance, angle, and temperature. In a high-stake environment, candidates are required to understand internal and external ballistics and be able to convert advanced theory applied to a practical setting, while using and employing advanced equipment efficiently. In addition, these judgments must be performed within a dynamic time frame, under pressure.

As a sniper pair, or as a single shooter, snipers need to be proficient in delivering precise long-distance shots from concealed positions, both at night and during the day. The ability to rapidly identify and acquire moving targets and shoot from several improvised shooting positions, both in an urban and rural environment, is also taught and assessed. To pass the course, the candidate needs to have a 70% pass rate on fieldcraft (navigation, stalking, observation skills, and combined tests where shooting is integrated) and an 80% pass on the shooting component of the course.

In the sniper course, there are 135 tests, 70 are different shooting tests, 65 tests are distributed between stalking, observation, judging distance, navigation, memory (KIMS), practical external ballistics, and shooting formulas (i.e., applied tables for windage, angles, and moving targets) and spotter (observer) tests. These tests are distributed throughout the course, while several are exactly the same and frequently repeated over the length of the course (Huse, 2020). The tempo of the program is high; 12+ h days are commonplace, and the sniper candidates' performance is assessed daily. These factors combine to make it one of, if not—the most demanding course for soldiers in the Norwegian Armed forces, and the course serves as the training pathway to becoming an operational sniper.

Therefore, reflecting these extremes of challenge, we were interested in examining the extent to which a performance psychology intervention, built on pre-existing grounds, as described, but with domain specific adjustments could be applied to this special military context (cf. Ytterbøl et al., 2022). To evaluate the impact of the performance psychology input, we examined outcome scores, reactions of participants, and perceptions of providers and used a 1-year follow-up to test the extent to which the knowledge conveyed had proven useful in the field.

## Methods

### Participants

Ethics approval from the University committee and local permission to conduct research in the Norwegian Armed Forces was granted. Prior to joining this course, all candidates are required to a pass a 3-week introductory program that is designed for conscript level marksman. The advanced education is an all-arms course developed for professional soldiers and non-commissioned officers, so that the basics of fieldcraft and small unit tactics are already well established. Since 2016 the number of SCs on the course has varied between 10 and a total of 14 participants on the 2020 course. In total, all 14 sniper candidates (SCs) on the 2020 course volunteered. To reflect the variation in the different units across the Norwegian military personnel, the purposive maximum variation sampling (Bryman, 2016) was carried out by the head instructors. This ensured a realistic proportion for participants from different units. In addition, it had the effect of reducing the number of potential participants to eight to conduct the in-depth interviews within an acceptable time frame. Participants were coded *Foxtrot, Golf, India, Lima, Mike, Sierra, Victor, Zulu*. They were aged 20–26 (m = 23.7) years in service ranged from 2 years to 7 years (m = 5.5 years); three respondents had already been deployed on combat duties.

In addition to the recruitment of SCs, the instructors, all of whom are subordinate to the sniper course leader, were also approached to participate. Unlike the participants, the instructors were performing military roles assigned to them by their senior commander. Once again, however, participation in the study was entirely voluntary. Assurances were provided to confidentiality, and all participants were issued with an information sheet and an informed consent protocol which contained contact instructions for the first author. All four instructors (*n* = 4) opted to participate (*blue, red, gray, and gold*) [Aged 30–35 (m = 33.5)].

## Procedure

### Research design

**Figure 1:**
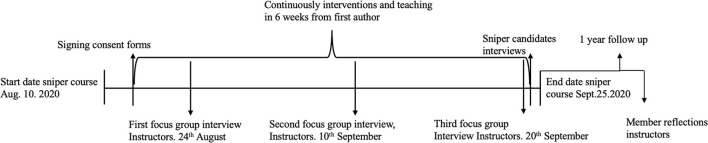
Overview of the flow of the sniper course in 2020.

An exploratory case study methodology was selected to provide a rich in-depth account of the real-world phenomenon within a specialized context. This approach was adopted to draw pertinent information from multiple sources through separate, though interdependent, sources of data (Willig, 2013; Bryman, 2016; Robson and McCartan, 2016; Yin, 2018). Data were analyzed with explorative reflexive thematic analysis (Braun and Clarke, 2022). Furthermore, outcome scores from present and past courses were obtained from NAF. In addition, member reflections were employed to enhance trustworthiness (Koelsch, 2013; Birt et al., 2016; Motulsky, 2021), while a 1-year follow-up to understand more about the perceived effects and retention of the MST curriculum was conducted (Figure 1).

We wanted to understand more about their learning experiences (Lachman, 1997) and how effectively these had been utilized when the participants deployed—or in some cases embarked on new careers. After the follow-up was completed with the snipers, the complete study in its final stages, with the follow-up of the snipers (Birt et al., 2016), was then presented *via* email and the instructors were invited to voice their opinion on whether the study resonated with their own experiences and to comment and ask questions they might have. This also gave the instructors time to test out what they picked up from the intervention and how it worked. Input was obtained through individually arranged telephone calls, so they could voice their opinions of the questions presented.

## The mental skill training intervention

As presented in the introduction, current research and applications of MST are plentiful and the effects point in a positive direction. However, there is a gap in research on “which aspects of MST are most relevant to the military” (Jensen et al., 2020, p. 11) and in addition, a lack of bespoke solutions for elite units and SOF in particular (Ytterbøl et al., 2022). Therefore, the authors wanted to present a bespoke approach to this special context presenting the overarching philosophical approach and how the MST intervention, based on pre-existing grounds, has been designed and delivered.

### Philosophical overview

The present MST intervention was designed to be in-keeping with the concept of adaptive skill, termed as the “the essential ingredient of developing expertise” (Ward et al., 2018, p. 2). Our goal for each SC was for them to acquire pertinent MST knowledge and to develop a deep understanding as to why they may consider using specific, performance enhancing MST techniques. In partnership with the training cadre, we sought to provide performance options rather than “solutions”. Explicitly, and as presented to all participants, we wanted the SC to discover and develop their own interpretation of the MST techniques by presenting them in multiple ways (Spiro et al., 1991). The use of this overt approach was well received by the participants, enabling them to learn the basics of the mental skills techniques, apply it for themselves and evaluate what they did, and further refine the MSTs employed to meet their own needs. To individualize the content of the MST training package, each participant was encouraged to employ the “test, tweak, repeat” philosophy (cit. Collins and MacNamara, 2022), to thus ensure that the functional complexities of the curriculum were preserved (Ward et al., 2018).

Moreover, as part of the present intervention, we elected to define the SCs on the expertise spectrum, regarding them as pre-elite (Collins and MacNamara, 2022). This is important as the approach stands in contrast to a competency-based model of skill development and enabled the research team to draw upon knowledge from the field of talent development (TD) in sports. Implicit in this decision is the acceptance that the development of expertise is likely to be non-linear and, at times, distinctly “rocky” (cit. Collins and MacNamara, 2012). Expanding on this, the overarching idea was for the SCs to be “expertise equipped, rather than just competence equipped” (Cruickshank et al., 2020, p. 240), implying it was not about giving the SC solutions which they are required to mimic but rather, to provide them with an opportunity to learn and develop their skills continually throughout their military careers. More specifically the first author utilized dialogical learning (Eikeland, 2008; Bardone and Bauters, 2017) as the starting point to generate an epistemological chain that facilitated the implementation of professional judgment and decision-making (PJDM) (e.g., Grecic and Collins, 2013; Collins and Collins, 2017).

### The mental skill training approach

Context is important, and the “effective use of mental skills requires application of such skills in scenarios where the skill is needed” (Hamilton et al., 2020, p. 130), an approach which we implemented in this case study. In planning and preparing the MST interventions, we focused on developing a program that enabled us to teach the mental skill techniques that have a research basis (see Table 2) and drawing from previous research, in particular the “personal performance plan” concept (PPP) (DeWiggins et al., 2010, p. 462). We broke this down to the following procedure: *preparing* is being able to develop the specific mental and tactical attributes needed to perform, *performing* is to develop key factors for performance, and *integration* is to accept the performance (optimal/sub-optimal) leading into *learning* and stimulating further development. This concept was established to aid the SCs perform on all aspects of the course. In addition, lectures were practically oriented and focused on the inevitable challenges that arose from the course, and situations of adversity commonly encountered by an operational sniper. All initial lectures followed the same format, a brief session on what the technique is, and how it is used, practice followed by a round of questions and answers. This enabled the SCs to possess sufficient knowledge to start their learning journey and implementing that specific technique.

**Table 2:** Overview of mental skill techniques on the sniper course.

**Type of skill**	**Description**
Goal setting	Adopting a pragmatic approach, where we focused on using process, performance and outcome-based goal setting using the (Specific, measurable, achievable, relevant, timely, evaluate, re-adjust) SMARTER structure (Locke and Latham, 2006; Kolb, 2015; Healy et al., 2018)
Imagery	Understanding the basics of imagery through the (physical, environment, task, timing, learning, emotion, perspective) PETTLEP model (Holmes and Collins, 2001; Lu et al., 2020)
Breathing	Learning the basic principles of applied diaphragmatic breathing (Ley, 1994)
Positive self-talk and affirmations	Learning the basics of how thoughts can influence behavior, learning to become conscious in how the sniper candidates internal dialog can increase or decrease performance (Hardy, 2006)
Relaxation protocols	The SCs were provided with a very basic understanding of relaxation protocols employed when using mindfulness (Hoyt, 2006; Meland et al., 2015). The first author presented examples of three 5–10–20 min applied mindfulness sessions, and recommended that the protocols, or parts of them were used before candidates went to sleep, or in downtime between lectures and training.

The MST training consisted of the skills listed in Table 2 and the overall lectures in Table 3.

**Table 3:** Classroom lectures presented on the sniper course in 2020.

**Date**	**Time**	**Subjects covered**
August 11.	30 min	Introduction to performance psychology and mental skill training.
August 12.	2 h	Goal setting in the context of the course. Developing generic demands for performance as a sniper and individual areas to focus on.
August 14.	1 h	What is stress and how does it affect us? (film produced by first author)
August 18.	1 h	What have you learned in the first week? Revisiting goal setting, then the MST techniques.
August 19.	1 h	MST curriculum (see Table 2).
August 21.	2 h	Relaxation skills and practice.
August 23.	1 h	MST curriculum (see Table 2).
August 26.	1 h	MST curriculum (see Table 2).
September 2.	45 min	Learning as an experiential process, accept the process.
September 3.	30 min	How to prepare for a test using MST.
September 4.	30 min	Guided mental rehearsal, learning about PETTLEP (see Table 2).
September 8.	1 h	Stress lecture repetition and sharing of experiences in the group.
September 9.	1 h	MST curriculum (see Table 2).
September 10.	1 h	MST curriculum (see Table 2).
September 11.	1 h	MST curriculum (see Table 2).
September 14.	1 h	Questions and answers on mental practice.
September 17.	1 h	Revisiting individual demands of the sniper. Individual progress and difficulties.

In the education component of the course, the first author also covered cognitive therapy's ABC model (Early and Grady, 2017), with a special emphasis on participants' behavior. Focusing on the behavioral aspect of their actions, participants were asked to consider what behaviors they manifested when they performed at their best; they were then encouraged to replicate these—in specific circumstances. The first couple of weeks spent working with this cadre detailed the basics of MST, usually in a classroom environment, before the course curriculum for the day commenced. The focus was on introducing the performance psychology curriculum. Making use of the 7-week course, an experiential learning approach (Kolb, 2015), where most of the task is known up front, was formulated to teach the sniper candidates about stress and stress responses and methods of coping. The first step was to repurpose a video the first author created to teach military cadets about stress—a practical explanation of the cognitive activation theory of stress (CATS; Ursin and Eriksen, 2004). This was followed up by a group lecture on coping responses to stress, how to understand one's own behaviors to mitigate against negative experiences by accepting stress as part of the learning journey. The rest of the course was focused on repetition and expansion of the MST techniques, based on dialog and ensuring a specific link to each SC's prior experiences. In total, the intervention comprised approximately 18 h of classroom lectures (see Table 3). In addition, the first author was present in the practical sessions during the course, making himself available for questions relating to MST, while at the same time providing candidates with the necessary space to reflect on their learning (Kolb, 2015). Therefore, no advice or solutions on specifics issues were provided by the first author at any stage of the course. When a question was forthcoming, where an answer could have provided a solution to the problem, the first author reframed it into a question to assist in the learning process, adhering to the overarching philosophy of this case study, especially focusing on learning as a conflict between what you already know—and the unknown—thus promoting self-efficacy (e.g., Bandura et al., 1999). In sum, we were endeavoring to support them, while seeking to afford candidates a learning experience. With reference to Table 1, it must be recognized that in combat split, second decisions have to be made, and the focus of the MST training should replicate this requirement, especially developing their own decision-making process, or else this MST intervention would be sub-optimal and potentially unethical: training for operational capability is ultimately the goal.

## Interviews

The exploratory case study and interviews were all conducted by the first author between August and September 2020. The interviews lasted between 30 and 60 min for instructors and 45–70 min for SCs. Each interview (with all participants) was conducted in an informal, though private setting. This allowed the interviews to be structured in a way that enabled respondents to be guided, so that their observations and thoughts emerged but, at the same time, provided them with the flexibility to talk freely (Braun and Clarke, 2006).

### Sniper candidates

To develop the semi-structured interview questions, the first author used his own military experience as well as capitalizing upon informal discussions with highly experienced snipers to further refine the semi-structured interview schedule. Following this pilot study, SC's interviews (*n* = 8) were conducted at the end of the 7-week course. Each interview was divided into three phases, commenced with an outline of the format, and then progressed by requesting that each sniper pictorially represents their experiences of the course on a graph. This representation included the individual's self-perceived most salient moments—both positive and negative.

The second phase of the interview focused on training prior to the course. This phase focused specifically on whether MST had featured in each candidate's preparation and, if so, how this was made manifest. The third phase focused on each candidate's perceptions of the sniper course, their performance development, and their use of the MST material that was presented. Due to the flexibility of the interview format, thoughts and meanings arose as the discourse unfolded (Braun and Clarke, 2019). Exemplar questions for the snipers included the following:

- “How did you experience the mental skills training during this course? What method (s) worked best for you?”
- “Related to the graph you've drawn, to what extent did mental skills training help you perform?”
- “Related to performing under pressure: to what extent did the course change or develop your mindset?”
- “In hindsight, what is your most valuable experience on this course?”

### Instructors

Interviews with the instructors (*n* = 4) occurred on three occasions (Figure 1) in a focus group format. Interview topics focused on performance under pressure; cognition and behavior experienced under pressure; and mental skills used in training and operational settings. The repetitive nature of the questions posed made it possible to go into depth in each area, and the informal group setting made it possible to talk freely and reflect upon what other instructors experienced. Exemplar questions for the instructors included the following:

- “What do you see/notice/hear from those who perform best under pressure?”
- “How can you contrast this with candidates who seem to perform poorly under pressure?”
- “Over the last week, in terms of shooting/performing under pressure, what have you observed?”
- “In which way did you observe or hear anything from the candidates/snipers which suggests they are making use of mental skills whilst performing under pressure?”

## Data analysis

All interviews were transcribed verbatim in Norwegian, before a reflexive thematic analysis (Braun and Clarke, 2006, 2019; Byrne, 2021) was conducted. After the final themes were actively generated, the translation process commenced taking extra steps to ensure the core meaning was transferred into English. This then enabled the other authors, as critical friends to review the content and offer constructive criticism on the themes identified (Smith and McGannon, 2018).

## Integrity, trustworthiness, and rigor

In this investigation, the first author enacted several roles: he is a former sniper, instructor, and Non-Commissioned Officer with over 20 years' experience soldiering in the Norwegian Armed Forces. He is also a performance psychologist in training, with a master's degree in applied coaching and currently undertaking a PhD education in applied performance psychology. The quality of the delivery was supported through the planning and execution process in the research team.

It is necessary to consider the potential for biases in relation to the present study (Levitt et al., 2017). The authors are interested in the potential of the psychological material utilized to improve the performance and wellbeing of the present set of sniper recruits—and potentially those that follow. As researchers, we are committed to analyze and present the data according to the intentions of the participants. However, as the first author makes his position clear in relation to the data and the participants, his subjectivity and the tensions that result from having a dual role in the present study are of note. Being both a practitioner interested in the efficacy of curriculum change, while also acting as a researcher designing the methodology and evaluating the evidence, can be viewed as a vital resource and asset as well as offering a potential confound (Gough and Madill, 2012; Braun and Clarke, 2019).

Reflecting this potential negative, several steps were taken to ensure methodological rigor and enhance trustworthiness. For example, member reflections, where respondents are allowed to read and comment upon either parts of the analysis or their interview transcripts, are often mentioned as the gold standard (Koelsch, 2013; Birt et al., 2016; Motulsky, 2021). The use of a long-term follow-up was another positive step in this direction.

## Results

We examined outcome scores, reactions from participants, and the perceptions of providers and used a 1-year follow-up to examine the extent to which the knowledge conveyed was useful in the field. For all qualitative data, the six phases of data analysis were conducted flexibly and systematically, allowing for lateral movement between stages (Braun and Clarke, 2019). In the first stage, the first author listened to each interview in its entirety on two occasions prior to commencing transcription. At this stage, cursory notes were recorded regarding salient details that occurred to the first author. These reflections were logged in a reflexive journal for the entirety of the study. In the second phase, the first author printed out interviews and placed them on a whiteboard, using marker pens to underline selected quotes and then coding them. Codes were then assembled into associated clusters. Based on each cluster, themes were generated as meanings became apparent across the data. Interestingly, both explicit and latent meanings were discovered which required that the respondents' intended meanings were considered against the first authors' impression of what was envisioned.

### Outcome scores on the sniper course

Naturally, there are statistics on the shooting results and pass rates from previous sniper courses. These are course results kept by the sniper section. Notably, the course has changed over the years, with regard to the classification of the different levels describing the sniper, training manuals that evolve and with equipment enhancements for the snipers. In addition, instructors with different pedagogical approaches and experience naturally influence the results. Therefore, direct comparison of results and scores carries some problems. Nevertheless, the statistics present the results and the 2020 sniper course was the first time MST was included. Furthermore, the demands to pass the course remain the same throughout these years, and the tests are the comparable. The candidate needs to have a 70% pass rate on fieldcraft, and an 80% pass on the shooting component of the course. In the sniper course, there are 135 tests, of which 70 are shooting-based (Huse, 2020).

As an important note, in the year of the intervention, 2020, for the first time, all SCs passed the course (For the years 2012, 2015, 2019, no comparable data exist). Furthermore, there seem to be some important variation in performances on the different components. For the years 2016–2020, those for which we have data from the shooting portion of the course, shooting scores seem relatively unchanged with 77% in 2016, 84% in 2017, 86% in 2018, and on the 2020 course with MST integrated 85%. The mean pass rate in the shooting component is 83%. However, for 2008–2018, the mean pass rate (shooting scores and fieldcraft scores) for the course is m = 43.2%. The lowest pass rate is 27% in 2016, and prior to the year of the intervention in 2020 (with a 100% pass rate), the previous highest pass rate is 67% in 2017.

From 2008 to 2018, Figure 2 indicates a high degree of variability year on year. Furthermore, given the emphasis of the curriculum during this period was on a technical/bio-mechanical model of skill execution, meaning MST was not actively a part of the curriculum. This can be characterized by a high, though variable level of course withdrawal/fail as the mean pass rate is 43.2%. Of interest, in the year of the intervention—where MST was taught and practiced—no course fails were recorded. If coincidental, it is at least a happy one!

**Figure 2:**
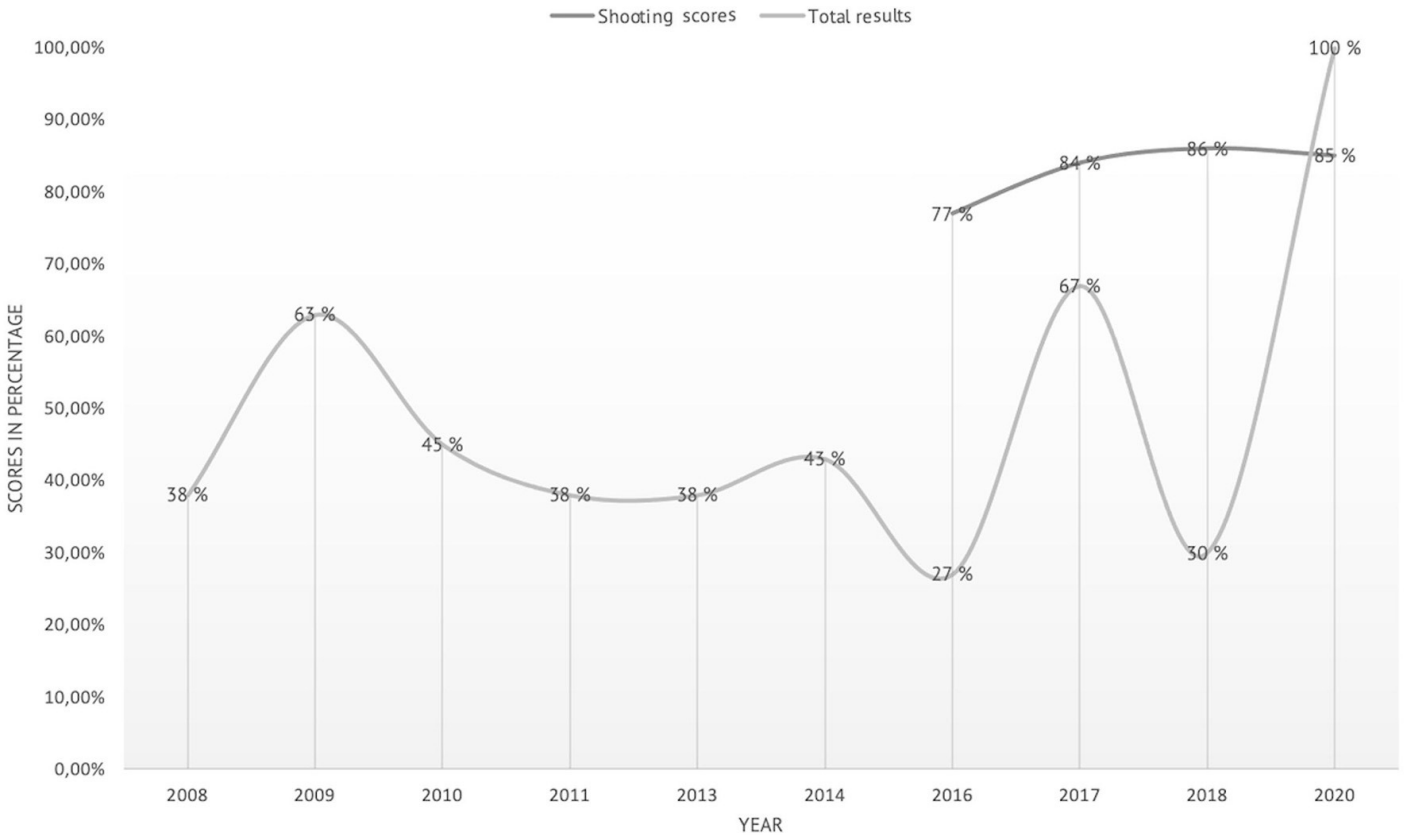
Overview of shooting scores and total results (passrates) 2008-20.

### Reactions from sniper candidates

The first key theme developed (Table 4) From the thematic analysis describes how the SCs experienced the MST taught on the course. First, respondents highlighted how it had assisted them in developing their understanding and subsequent individual approach of using MST to improve their performance. Second, it became apparent how the mental techniques, especially breathing and imagery, had been adopted by all respondents as effective tools. Notably, however, the SCs developed their individual versions of utilizing these techniques as exemplified by Foxtrot:

Just saying to myself: here you got to get a f^*^ grip of yourself. It is like a growl. Now, I am doing it like this and this. More talking to myself than visualizing it. I'm doing this, then I walk through it step by step. I was aware of it on one of the tests. My performance started to decline. I did not feel like it was my nerves, I had to speak to myself [knocks his knuckles on the table]. I felt like I got a boost again. Then I performed well.

**Table 4:** Key theme: key positive benefits (*n* = 8).

**Subthemes**	**Raw data exemplar**
Developing a system (*n =* 8)	“It has enhanced my thoughts and experiences I had from before. But now I have a deeper understanding of it, and it has become a more usable tool than before. Things I used to do from before was put into a structure.”
MST used (*n =* 8)	“What worked best for me is imagery, at least those tests I have experience with from before. On the tests I don't know or have little experience with, imagery has not worked for me… When I have managed to take control over my breathing, I have performed better than I thought.”
Positive impacts (*n =* 8)	“This supplement to the course, the mental stuff, belongs very much on this course. It has helped me to relax more maybe. And even if I'm not quite there yet with all the stuff you are talking about, I want to get there, and I have a strong belief that this works."

Foxtrot uses self-talk combined with affirmations. It not only prepares him for action but also helps him refocus when he experiences his performance declining. All the SCs utilize their own system. Lastly, they all agreed on the importance of integrating a performance psychology package in this type of education, although it may not provide immediate results. An example from SC India is as follows:

It has enhanced my thoughts and experiences I had from before. But now I have a deeper understanding of it, and it has become a more usable tool than before. Things I used to do from before was put into a structure.

He describes how he has developed his understanding and through his learning process it has become a tool that is more available to him. Furthermore, he exemplifies the following:

It has helped me distinguish between the factors I can influence and not. Changed my focus on what I spend energy on. It's tempting to say, my gun is off, I have a bad day. I force myself to think what I do wrong here. This is where I need to focus (India).

In SC India‘s example, it seems that through his experience of a deeper understanding, he is to a larger degree capable of maintaining focus through an active choice.

The second key theme (Table 5) that was generated also consisted of three subthemes.

**Table 5:** Key theme: performance under pressure (*n* = 8).

**Subthemes**	**Raw data exemplar**
Approach to pressure changed (*n =* 8)	“It is the amount of pressure you put on yourself. It's like, I'm not going to be worse than anybody else. Just get into it and do what you have done 1,000 times before. Nothing new, but just focus on the tasks ahead. I think I was more result oriented before, but now I focus more on the process itself.”
Increasing self-confidence (*n =* 8)	“My self-confidence has improved, not necessarily because I am a better sniper, but because I have changed my attitude toward performance.”
Developing procedures (*n =* 8)	“The first thing I do in the morning is attaching (sic) my suppressor, find my bean bag and hearing protection, and while I walk to pick up my ammunition I start on my breathing process, so, I feel myself calm down while I fill my magazine. Then I stand behind the gun, move into position, feet apart, move into my gun, get it into my shoulder, all of this while I breathe calmly, take off the scope protection, adjust the beanbag, while I breathe ouuuuuuuuut. I am mentally in a calm state. Then I am ready.”

It seems that the SCs became more aware of how pressures influenced their performance and what they could individually do to mitigate it. Understanding their individual stressors is described as an important step to increase their performance.

SC (Sierra) describes how the MST training helped cope with this pressure, when he had to retest one of the examinations to be able to pass the course:

I have learned a lot from that breathing stuff. I think it saved me when I had to retest one of the final exams. I had this one test: it's been my Achilles heel. So many thoughts running through my mind, doing dry fire practice before we get the order to load our weapons, completely calm. But then my heart immediately–it starts to pound, and my reticle [what you see in the riflescope] shakes all around the place. So, breathing and where to put my mind during performance.

SC Sierra especially highlights that even during extreme pressure, he was able to utilize his own version of mental skills, and assist him in not only coping, but also performing.

Furthermore, the SCs especially identified the aspect of self-confidence and how important this is as a psycho-behavioral skill. Golf describes his experience:

You hit spot on (*sic*.) in one of your lectures, on identity. Because this course means so much to me that it has become my identity. And then when I doubt myself, I don't hit that target.

Lastly, all stressed the ability to develop their own set of performance enhancing procedures to be able to focus cognitively on important aspects for their performance.

### Perceptions of instructors

Table 6 the instructors had been able to focus on observing the candidates over a long period of time. Interestingly, they collectively agreed that the MST package had improved the SC's performance, through the SC's individual applications.

It's hard to say what puts the SC in the top and further down the list, but what I can observe is the SC who takes the time to do breathing exercises and imagery before a task, performs better (Blue).

**Table 6:** Key theme: different strategies (*n* = 4).

**Subthemes**	**Raw data exemplar**
MST observed	“I feel, it was yesterday when I thought about it. I observed on the Cold Bore Level test [one of the tests that are considered very demanding because of the repetitive nature] and for the first time I heard and observed that the guys were doing those breathing techniques. It was obvious yesterday that they breathed very well. I have not noticed that before.”
Learning and confidence	“Confidence in one's own skills, awareness of one's own limitations, do not blame everything else, instead acknowledging that this is a learning process. For example, it wasn't a 100 (score) here, I misjudged the wind, but I observed and recorded that the wind can be like that. Learning. Tools in the box. Some (SC's) are very different there. Some are just, s^*^. f^*^. While some are like, oh can it be like that too, that openness. It's just to acknowledge that experiential learning, I must (go through) do that to learn (wind). Someone excels with a good ability for it. Embrace the learning as opposed to other things.”
Introspection	“They can lose control, but those who seem do the right things has a higher baseline (of performance). Maybe one bad result, but the total results are better. They take the defeat as a way to learn on an equal footing with the fact that they were praised for doing well, for example, now it went well, the feedback has been taken, the same mistake rarely happens twice in a row. In a way, they take the defeat as learning in the same way as sitting in the classroom and getting a lesson on ballistics or whatever. And learn something from it, instead of thinking, now it's gone to h^*^, now we're on a bit of a slippery slope in a way.”

At the same time, it seems that accepting that the course and subsequent experiences were a learning process acted positively on the SC's overall confidence, perhaps through accepting that it is about a learning process.

One example is a sniper candidate who has claimed ownership and shows it through his actions. He has taken the feedback and done something with it. And it shows, both in his scores and his behavior. His results are his own doing (Blue).

Furthermore, based on instructor observations, the way the top performing snipers were able to reflect, evaluate, and adjust their actions seems to be imperative for their overall results.

When it comes down to the final theme (Table 7), several interesting factors were developed.

**Table 7:** Key theme: optimal vs. sub-optimal performance (*n* = 4).

**Subthemes**	**Raw data exemplar**
Attentional control	“They guys who perform well on the evolutions are the ones who can focus on the tasks they have ahead of them, and not the prerequisites.”
Cognitive overload	“I just think, that's just the way it is with some people, some get tired faster than others and... others just, what can I say, give more of a d^*^mn and don't think about it so much, while others think about it more. Now I'm tired, now I'm bored. Now it's almost over. I think it is individual, yes.”
Developing a combat mindset	“To be able to correct quickly, to correct for a miss, why is it important, I used the example then that we don't train to lie down shooting steel targets, that's why, we train to shoot other people, what is natural then is, when you shoot you move, shoot back or do something else. Therefore, it is in addition to for example wind adjustments, to be able to make corrections quickly. When I used it as an example, it seemed as if a light switched on for several of them.”

Clearly, attentional control is a performance indicator. Being able to direct focus and maintain it is observed and agreed upon by the instructors as being an important factor.

Furthermore, the ability to accept responsibility for their own performance was an important factor, together with how the SCs approached the mental load this course presents.

For example, one SC noticed that his steel target is a bit perpendicular to his position, and he puts all his effort into that issue instead of focusing on hitting the target. Another SC, at the same target, doesn't make a big deal, just pling, pling [steel target resonance] and passes the test (Gold).

Lastly, the ability to understand what they are training for is operational capability seems to be a very important factor to achieve high performance on the course. As the instructors agree on the fact that being able to mentally put yourself [the SCs] in the situation and context that comprises a sniper's job description. Perhaps the development of what can be described as a combat mindset of what the job entails creates an achievement motivation to perform at the course, to be prepared for the job.

### Member reflections and 1-year follow-up

#### Sniper graduates

Such a long gap is uncommon in qualitative analysis but, we would suggest, essential in cases such as this where people are prepared for challenging contexts. In short, they are only able to comment on the impact of their new skills when these have been field tested. Of the snipers, five out of the original eight respondents opted to answer. The first questions were “Now, 1 year later, what, if any mental skills techniques do you still use?”.

One sniper had been deployed and, as such, had tested his skills from training in the most difficult situations:

When the rockets landed around us, and the counterattack systems thundered like he^*^, I used a breathing technique to not get too influenced by the surge of adrenaline. It kept me at a level where I was focused and “switched on”, but not in the black zone.

When we got to the area of the rocket impact and I had to sit down to treat people, I used visualization techniques. Gather the information I had about the place and imagine the worst possible scenario. Create a mental picture of what may meet me at the point of impact [of the rockets] (India).

Interestingly, the other snipers described employing a combination of skills from the performance psychology curriculum.

Mainly it is the breathing techniques that I have taken with me and that I use actively. It helps me both with the purely physiological aspect of calming my heart rate and reducing my stress by shifting my focus from external factors that cannot be influenced to the breathing that can be influenced. Otherwise, I use a lot of visualization when training individual skills. This happens quite unconsciously and without me having any system behind it (I imagine before I do) (Mike).

The graduate snipers used their learning both in performance scenarios but also in life in general.

“In addition, self-talk as a confidence builder has become a bigger part of life, both in and outside of work” (Zulu).

Over to the next question: to what degree do you think the integration of the MST contributed to the results on the course?

“I think the mental training has had a big impact on the pass rate of this course and question the fact that this is not part of a specialist's sniper kit, as a standard” (Mike).

The other respondents voiced similar opinions. They described how parts of the performance psychology package assisted them with performance on the course, especially in critical moments, maintaining or regaining focus and performance, and that they collectively agree that the course was perceived holding a higher standard with this addition.

I think it is difficult to say to what extent the mental training affected the pass rate. I know for myself that I got better scores on several shooting exercises due to the mental tools we were given, and I think the course as a whole was much better because of the mental training (Mike).

#### Instructors

They universally agreed that the integration of performance psychology techniques was a positive and necessary contribution to optimize not just the course, but the eventual performance on operations, too. They highlighted the positive effect on talent development they experienced when the focus was placed on the overall learning process, not just the results. In addition, Gray explained “That the research made practical sense and when I read the interviews with the snipers, I recognize my own mindset.” He stated that he wished he would have had the MST tools when he went through the course. Red specifically highlighted: “It was very interesting to read and matched my own perceptions of what was going on.” Lastly, all instructors highlighted the experience from Iraq, with India as an excellent example of real-life skills employed. They were reassured that he was able to utilize the skills he learned on the course.

## Discussion

Table 8 presents an overview of the qualitative findings. First, and against our primary objective, it is important to recognize that the intervention showed positive outcome on all four measures used: quantitative data from the results, interviews with SCs, interviews with instructors, and the follow-up. Moreover, the SCs perceived MST as a valuable contribution to the course. Indeed, each SC pinpointed the type of situations in which he might use a certain mental skill, based on their individual approach and learning experience.

**Table 8:** Overview of qualitative findings.

	**Key themes**	**Subthemes**
Sniper candidates	Key positive benefits	Developing a system, MST used, positive impacts
	Performance under pressure	Approach to pressure changed, increasing self-confidence, developing procedures
Instructors	Different strategies	MST observed, learning and confidence, introspection
	Optimal vs. sub-optimal performance	Attentional control, cognitive overload, developing a combat mindset

Looking at the data and compared against the most recent courses, a pass rate of 100% was accomplished in the 2020 version with MST integrated in the course. Extracting the shooting data only, the results are stable over the years (2016–2020), also when MST was included in the curriculum. When the significant component of fieldcraft is included, the comparable data (2016–2018) display a mean pass rate of m = 43.6 % on the course. There are too many variables in an uncontrolled environment (instructors, manual, and equipment changes) to draw any definite conclusions. However, shooting is a bio-mechanical skill which, if you practice enough, you get better at (Laaksonen et al., 2018). For a sniper, being able to perform this skill is of vital importance, but compared to sport-related shooting we would contend, with reference to Table 1, that the context is extreme. In the sniper course, the results pay tribute to the competency of the instructors in teaching their students. On the contrary, in fieldcraft and combined tests, where the instructors are not there to directly assist and teach, the SC has to rely more on their own decisions, related to self-confidence. The data can indicate the importance of the cognitive and metacognitive aspects (cf. Veenman et al., 2006), the requirement to think and perform under very high levels of pressure individually, is one area where the MST interventions have an impact as the SCs describe their developed performance under pressure. This can be explained by the fact that “Either you are capable of planning your actions ahead and task performance progresses smoothly, or you don't, and your actions go astray” (Veenman et al., 2006 p. 5). In addition, the SC's development of their own personal performance plan (DeWiggins et al., 2010) can enhance their metacognitive capacity. It is difficult to explain the exact patterns, based on the analysis it is a result of the context specific comprehensive MST approach rather than a single skill. For an operational sniper, the ability to maintain cognitive control cannot be overstated. Being able to make the right decision in extreme circumstances demands high levels of cognitive and decision-making skill: Each of the shots fired, or the shots not fired, requires to be justified (cf. Bar and Ben-Ari, 2005).

There is a clear pattern of how differently the snipers approach their own performance, enhancing the statement from Hamilton et al. (2020), that it is context-dependent. Furthermore, that each SC has their own method of approaching the challenges at hand. On the one hand, this could be directly linked to the philosophical approach of how MST was taught during the lectures, through the concept of developing adaptability (Ward et al., 2018). We explicitly focused on creating the building blocks to become experts in the field, focusing on teaching them the how and why of performance development, not just the what is or basic competencies (Cruickshank et al., 2020). Of course, this difference in approach could also be ascribed to the natural progression resulting from the course. However, based on the analysis, there seems to be a clear and positive relationship with what this cohort of snipers has been taught, how they developed themselves (Kolb, 2015), and how they increased their self-efficacy during this period (Bandura et al., 1999).

Based on the difference in prior military and MST experience among the SCs, it is natural that the ones with less experience have more to learn, and this can be describes as moving through stages on the novice-expertise continuum (Dreyfus and Dreyfus, 2005). Our claims for causation are reinforced by the fact that several SCs explain how they have had basic knowledge of the techniques from before but that, supplemented by the content of this course, they were able to build the skills into a system, more specifically their own system. This occurred even if they did not yet have the experience, or complete mastery of the mental skills.

Our approach, equipping them with the methods needed to become experts, is well supported by research (Cruickshank et al., 2020) and underlines the importance of a philosophical foundation to teaching these methods (Bardone and Bauters, 2017). Of course, since there were no control group, it is premature to draw any definite conclusions. However, based on the analysis, the large individual difference in what MSTs were used, and how participants contextualized it on this course shows promise toward creating a basis for teaching MST in this context.

Of relevance, each SC utilized different methods from MST and supporting instructor observations. Imagery, using the PETTLEP model (Holmes and Collins, 2001; Lu et al., 2020), was preferred among all SCs. In addition, diaphragmatic breathing (Ley, 1994) was stated as the most widely adopted and readily applicable MST covered. On the one hand, jumping to conclusions and extract imagery and breathing as optimal solutions and downgrade the other mental skills is a potential pitfall. As an example, the use of mindfulness-based approaches has a strong research base, also in the military (cf. Nassif et al., 2021). Based on the analysis, relaxation protocols (see Table 2) were not favored by the SCs in this context. At the same time that particular skill probably takes a longer time to learn and in an intensive sniper course, it is probably not the best place to measure outcomes. On the other hand, different relaxation protocols probably impact performance positively, if only through increased quality of rest and sleep. Based on the instructors' observations, supported by the SC's experiences, improvements in performance were perhaps due to more than just a single “skill”. It appears that a genuine change in mindset occurred, toward a combat mindset (cf. Boe et al., 2020; Smith et al., 2020) meaning one where participants can contrast perspectives and weigh up the optimum blend for each challenge, in the context of becoming a sniper, perhaps leaning toward achievement motivation, demonstrating a higher ability to perform (Nicholls, 1984). Following the ABC model “we don't see what it is: rather we see what we think it is” (Early and Grady, 2017, p. 43): This enabled the SC to actively make a choice on where to put effort regarding his performance. Interestingly, when the SCs experience doubt regarding their own capabilities, this leads to a downward spiral—degrading their performance and resulting in difficulty utilizing the mental skills they are familiar with but have not yet fully embedded. In contrast, and as a growing feature as the course progressed, SCs who act toward what they can influence, rather than ruminating and experiencing a dip in self- efficacy, are able to employ the mental skills more fully to be present in the moment, especially breathing (Ley, 1994). This in turn enables the SCs to maintain a higher degree of attentional control (Eysenck et al., 2007) and also retain a high level of “skilled intuition” (Cruickshank et al., 2020, p. 245), which is vital if decision-making is to be of a high standard in natural environments, following the recognition-primed decision (RPD) model (cf. Klein and Wright, 2016).

Finally, the follow-up data provide narrative evidence that learning has taken place, and skills were applied in an extreme setting and as described in the development of the interventions, namely “expertise equipped, rather than just competence equipped” (Cruickshank et al., 2020, p. 240). Self-talk was also put forth as developing confidence in wider aspects of life. Instructor comments supported the authors initial analysis and that MST has its place on a sniper course.

Of course, the study is not without its limitations. We have not used a control group, although comparisons to previous years on an almost identical course (minus the MST) do go some way toward addressing this. Similarly, much of the work is based on self-report data, and the expectancy effect of the interventions must be allowed for. However, triangulation of SC perceptions with performance data, instructor perceptions, and the delayed follow-up have hopefully gone some way to addressing this shortcoming. Furthermore, the relationships and challenges of working in pairs (cf. Orasanu et al., 2008) have not received any attention in this investigation. As a final note, the philosophy, pedagogy, and teaching MST in this course are not standard in the NAF, meaning the novelty of the interventions itself could have influenced their motivation and interest to perform. All these issues notwithstanding further research in this very challenging and unique context are certainly warranted.

## Conclusion

Acknowledging that testing interventions in a real-world context is challenging, this is also where elite soldiers and operator's train and where the elite soldiers and operators are expected to perform are even more “in the wild” (Chamberlain et al., 2012). Therefore, the authors believe that conducting context and culturally specific case study research on a small, special population is important to move the field of performance psychology further (Ytterbøl et al., 2022).

The present investigation describes a relationship between the implementation of a mental skill training curriculum and subjective improvement in snipers' performance. Data on almost identical courses up until 2020 are outlined and provide an indication that the integration of a performance psychology package with MST could improve performance. This contention is unanimously supported by the instructor's observations. Furthermore, the SC's descriptions of the performance psychology package provide an insight into the previously intangibles of developing a form of “tactical wisdom”, individual strategies of performance and decision-making. Interestingly, but not necessarily surprisingly, we saw that both the philosophy and methods of delivery for MST play an important role. Perhaps the key takeaway is that this context and culturally specific and comprehensive MST intervention shows positive results on performance development. Furthermore, adding a well-planned performance psychology package could improve the overall learning process through increased awareness and space for individual applications of the skills taught. It is not to function as some sort of “magic potion” that solves all problems, and it should be a natural part of their training and education.

For the sniper candidates specifically, the introduction and implementation of MST provide a positive impact on all performance markers associated with this course. The follow-up indicates that they also learned and retained the skills developed well enough to function in combat situations. On the outcome scores of the course, no conclusions can be drawn but the results were positive in the 2020 intervention. Further quantitative performance and psychometric data, including a control group, are warranted to understand more about what the optimum intervention model can look like. However, the authors believe that our research presents a concept that can be implemented and developed further.

## Data availability statement

The original contributions presented in the study are included in the article/supplementary material, further inquiries can be directed to the corresponding author.

## Ethics statement

The studies involving human participants were reviewed and approved by University of Central Lancashire BAHSS2 0111. General permission to conduct research on military personnel from the Norwegian Armed Forces has been granted, in addition to local permission from the Norwegian Army. The patients/participants provided their written informed consent to participate in this study.

## Author contributions

All authors listed have made a substantial, direct, and intellectual contribution to the work and approved it for publication.
